# c-MYC—Making Liver Sick: Role of c-MYC in Hepatic Cell Function, Homeostasis and Disease

**DOI:** 10.3390/genes8040123

**Published:** 2017-04-19

**Authors:** Kang Zheng, Francisco Javier Cubero, Yulia A. Nevzorova

**Affiliations:** 1 Department of Immunology, Complutense University School of Medicine, Plaza Ramón y Cajal s/n, Madrid 28040, Spain; robertzheng@163.com; 2 12 de Octubre Health Research Institute (imas12), Madrid 28041, Spain; 3 Department of Internal Medicine III, University Hospital RWTH, Aachen 52074, Germany; 4 Department of Animal Physiology II, Faculty of Biology, Complutense University of Madrid (UCM), José Antonio Novais 12, Madrid 28040, Spain

**Keywords:** c-Myc Avian Myelocytomatosis Viral Oncogene Homolog, hepatocellular carcinoma, alcoholic liver disease, hepatoblastoma, hepatitis B virus, hepatitis C virus, Liver

## Abstract

Over 35 years ago, c-MYC, a highly pleiotropic transcription factor that regulates hepatic cell function, was identified. In recent years, a considerable increment in the number of publications has significantly shifted the way that the c-MYC function is perceived. Overexpression of c-MYC alters a wide range of roles including cell proliferation, growth, metabolism, DNA replication, cell cycle progression, cell adhesion and differentiation. The purpose of this review is to broaden the understanding of the general functions of c-MYC, to focus on c-MYC-driven pathogenesis in the liver, explain its mode of action under basal conditions and during disease, and discuss efforts to target c-MYC as a plausible therapy for liver disease.

## 1. Introduction

*c-MYC* is a highly pleiotropic transcription factor known to control cell cycle progression, proliferation, growth, adhesion, differentiation, apoptosis and metabolism [1,2,3]. The *c-MYC* gene was discovered over 35 years ago [4,5,6,7,8,9] as the cellular homolog of the retroviral *v-MYC* oncogene that causes myeocytomatosis (leukemia and sarcoma) and since that time became, perhaps, one of the most studied proteins in the history of human biology.

The human *c-MYC* gene was first revealed in early studies of fulminant chicken tumorigenesis [7,9] followed by the finding that human *MYC* is consistently altered by balanced chromosomal translocation in Burkitt lymphoma [10,11]. These discoveries attracted much attention for *c-MYC* research, leading to development of the “*the oncogene from hell*” concept [12]. *c-MYC* belongs to the family of *MYC* genes that includes *b-MYC*, *l-MYC*, *n-MYC* and *s-MYC*; however only *c-MYC*, *l-MYC* and *n-MYC* have neoplastic potential [6]. Since the 1980s, *c-MYC* studies have focused on its role in liver carcinogenesis—hepatocellular carcinoma (HCC). The ability of *c-MYC* to promote hepatic tumorigenesis has been demonstrated not only in vitro and in vivo studies, but also in human cancer.

Indeed, the interest in *c-MYC* research in the hepatology field over the last three decades has continuously increased, based on citations in PubMed. Interestingly, the number of publications covering “*c-MYC*” are appearing at around eight reports per month in recent years. Moreover, new data suggest that *c-MYC* deregulation in liver disease is more common than expected, and not only restricted to HCC development, but it also includes many other chronic liver diseases, such as alcoholic liver disease (ALD). Therefore, the main purpose of this review is to: (i) link *c-MYC* with different types of liver pathology; (ii) describe the possible mechanisms of action; and finally, to (iii) discuss ongoing efforts in targeting the unique properties of *c-MYC* for the treatment of liver disease.

## 2. c-MYC Functions in Liver Regeneration, Health and Disease: How Important or Dispensable?

The transcription factor *c-MYC* has been strongly associated with hepatocyte proliferation occurring during liver regeneration. During this process, quiescent hepatocytes synchronously enter the cell cycle and undergo one, two or more rounds of replication in order to restore liver mass. As an immediate early gene, *c-MYC* is considered to be a key factor in the transcriptional response that leads to the transition of hepatocytes from G_0_/G_1_ to the S phase. The expression of c-MYC rapidly increases during the pre-replicative phase which precedes DNA synthesis within the first 30 min following partial hepatectomy (PH)—reaching its peak levels by 2 h, followed by a second peak, 8 h after PH [13,14].

Quiescent and proliferating hepatocytes from the regenerating liver contain similar levels of c-MYC protein. Hence, in quiescent cells, c-MYC is typically localized in the nucleolus, while PH induces its nuclear translocation. In addition, c-MYC is also localized in the nucleus in highly proliferating fetal hepatocytes. This evidence indicates that c-MYC localization is altered in close association with cell proliferation, while sequestration in the nucleolus prevents c-MYC-dependent activation or repression of essential target genes involved in liver cell proliferation and growth [15].

Germ-line deletion of *c-MYC* leads to multiple abnormalities and death, at day 9–10 during embryonic development [16]. To analyze exhaustively *c-MYC* function in liver regeneration, inducible conditional approaches were also used in newborn and adult transgenic (tg) mice. Perinatal inactivation of *c-MYC* in newborns in liver (mx-Cre^+^/c-MYC^tg^ system activated by injection of polyinosinic-polycytidylic ribonucleic acid (pIpC) two days after birth) caused disorganized organ architecture, reduced hepatocyte size and cell polyploidy. However, *c-MYC*-deficient hepatocytes proliferate normally suggesting that postnatal hepatocyte proliferation is a *c-MYC*-independent process [17]. In line with this finding, another study demonstrated that hepatocyte-specific conditional knockout mice using Cre-mediated ablation of *c-MYC* (alb-Cre^+^/c-MYC^tg^) is not required for normal hepatic development after birth [18].

Nevertheless, published reports on the effect of *c-MYC* depletion on liver regeneration following two thirds PH are not fully consistent. In one study, adult mx-Cre^+^/c-MYC^tg^ mice injected with pIpC one week before PH showed reduced proliferating cell nuclear antigen (PCNA) and cyclin A expression, two days after partial resection [17]. In contrast, Sanders and colleagues [18] recently showed that alb-Cre^+^/c-MYC^tg^ display slightly less numbers of hepatocytes in the cell cycle, 48 h post-resection, but absolutely no delay in liver mass restoration. Additionally, adenoviral Cre deletion in floxed *c-MYC* consistently reported total recovery of the liver mass, seven days after resection [19]. According to these studies, *c-MYC* is not only dispensable for hepatocyte proliferation after birth, but also for hepatic mass restoration during regeneration following PH. However, in developing tissues, *c-MYC* may play a pivotal role in cell proliferation such as in the hematopoietic lineage where deletion results in defective hematopoiesis and angiogenesis—leading to embryonic lethality [20].

Therefore, it seems that *c-MYC* is required for embryonic development but is in fact dispensable for hepatocyte proliferation and growth in the adult liver. Moreover, these studies indicate a cell-type and context-dependent role of *c-MYC* in hepatic proliferation. Altogether, these results highlight the intriguing physiological functions of *c-MYC* in the process of normal liver development, hepatocyte proliferation and growth.

## 3. Deconstructing c-MYC: Transgenic Models

Since deregulated c-MYC expression is an early event in carcinogenesis, the definition of the consequences of c-MYC overexpression in hepatocytes is of great interest. An early paper published in 1988 [21] already described that cultured hepatocytes electroporated with the *c-MYC* gene exhibited rates of DNA synthesis approximately 50% higher than those of untreated hepatocyte cultures. Importantly, this increase was dependent on the amount of *c-MYC* transfected with the DNA.

Reasonably, in order to reflect the role of c-MYC in hepatocytes in vivo accurately, different models overexpressing c-MYC were generated (Table 1).

Kim et al. [31] evaluated the effects triggered by transient overexpression of c-MYC using recombinant adenoviral transfection in order to deliver the human *c-MYC* transgene to murine livers. Ectopic expression of c-MYC caused hepatocyte hypertrophy as well as enlargement of nuclei and nucleoli of liver cells. Moreover, changes in liver cell size due to overexpression of c-MYC were accompanied by increased expression of genes encoding ribosomal and nucleolar proteins, albeit with no significant proliferation. Interestingly, c-MYC has been linked to ribosome biogenesis [32]. Altogether these data provided clear evidence that inappropriate overexpression of c-MYC confers a growth benefit to liver cells in vivo.

A considerable number of publications from the group of Prof. Snorri Thorgeirsson has largely improved our knowledge of *c-MYC* in HCC, and has essentially become the benchmark of experimental models of murine liver carcinogenesis. Does c-MYC overexpression in hepatocytes confer proliferative potential to livers of alb-Cre^+^/c-MYC^tg^ transgenic mice? This group [22] found that, at four weeks, when the liver is actively developing, the rate of regeneration in c-MYC transgenic mice was significantly greater than that of wildtype (WT) mice. Unexpectedly, the capacity of transgenic livers to regenerate dramatically changed once liver growth was achieved, after birth. Moreover, ten-week-old c-MYC^tg^ transgenic mice showed exacerbated cell-cycle progression and fast recovery after PH. The effect of the *c-MYC* transgene on cell proliferation was caused by a decrease in the length of the pre-replicative period (approximately of 10 h), exacerbation of the G_0_/G_1_ transition and an increase in the synchrony of cell-cycle progression [22].

A high rate of hepatocyte proliferation leads to ineffective DNA repair though, increasing the frequency of mutations due to replication errors and, consequently, to increased cancer susceptibility. Indeed, hepatocyte-specific alb-Cre^+^/c-MYC^tg^ mice are predisposed to HCC [23,33]. Therefore, 40% of c-MYC^tg^ mice display tumors at 45 weeks of age, whereas 80% of 65-week-old c-MYC^tg^ mice exhibit HCC [24]. Appearance of benign hepatic neoplasms occurs in eight-month-old c-MYC^tg^ mice, while HCC develops at the age of ten months. The histological type of HCC observed in c-MYC^tg^ animals can be either trabecular or solid, ranging from well-differentiated to poorly differentiated tumors associated with cell polymorphism, atypia and areas of haemorrhagic necrosis.

We and others have reported that alb-Cre^+^/c-MYC^tg^ mice exhibit excessive levels of the tumor-suppressor p53, essentially involved in the induction of DNA repair and down-regulation of intracelular levels of ROS [34,35]. We suggest that high p53 levels contribute to the particular hepatocarcinogenic phenotype of alb-Cre^+^/c-MYC^tg^ mice characterized by slow-growing liver tumors, high latency and low malignancy [23]. These tumors finally become confluent and replace most of the hepatic parenchyma [25].

Subsequently, the interaction between growth factors and c-MYC during malignant transformation in the liver occurring in c-MYC^tg^ animals was analyzed. This crosstalk appears to be essential for the different outcomes of the hepatic neoplastic process. For example, co-expression of c-MYC and transforming growth factor-alpha (TGFα) as transgenes in the mouse liver resulted in a dramatic acceleration of neoplasia as compared with the expression of either of these transgenes alone. At 17 weeks, 20% of these mice display HCC consisting of multiple foci of carcinomas and adenomas, which affect 100% of the mice at 40 weeks of age [23,25,26,36]. Another example of the crosstalk between c-MYC and growth factors are double transgenic mice expressing epidermal growth factor (EGF) and c-MYC, in which exacerbated tumor progression and mortality led to the occurrence of HCC in 100% of the mice after approximately 12–18 weeks [36,37]. Altogether, the rapid HCC occurrence in the double transgenic models, compared with the parental lines, suggests that the crosstalk of c-MYC and some growth factors increases malignant transformation via selection and expansion of preneoplastic cells [36].

A more aggressive tumor phenotype is also found in double transgenic mice, c-MYC and E2F transcription factor 1 (E2F1). In addition to sharing functional properties, increasing evidence suggests that these two proto-oncogenes can reciprocally regulate the activity of each other. Indeed, the requirement of distinct E2F members for mediating c-MYC-induced proliferation versus apoptosis has been demonstrated [38]. Indeed, the cooperation of *c-MYC* with E2F1 is necessary for the expression of target genes throughout the cell cycle [39,40,41]. Furthermore, it is very likely that survival of c-MYC-over-expressing cells is dependent on E2F1 activity, suggesting that E2F1 sustain abnormal c-MYC-driven cell growth via suppression of c-MYC-induced apoptosis [38,42,43]. Alterations in the expression of c-MYC and E2F1 affect liver cell ploidy during hepatic growth—after birth and before tumor onset. However, their functions are different. E2F1 stimulates the proliferation of diploid cells, characteristic of preneoplastic stages of HCC, while c-MYC functions to accelerate hepatocyte polyploidization related to age [27].

In sharp contrast, co-expression of Hepatocyte growth factor (HGF) and c-MYC in the liver delays the appearance of preneoplastic lesions and prevents malignancy. This finding is of particular relevance since HGF selectively inhibits proliferation of transformed hepatocytes, stimulating the proliferation of normal hepatocytes and acting as a powerful tumor suppressor [25].

Not surprisingly, lack of the molecular gatekeeper p53 accelerates HCC in c-MYC^tg^ in the liver [28]. Enhanced tumor growth, pronounced malignant structure and an invasive growth pattern correlated with p53-deficiency in livers of alb-Cre^+^/c-MYC^tg^. Additionally, introduction of the p53 null alleles into alb-Cre^+^/c-MYC/IgEGF^tg^ double transgenics caused postnatally abnormal hepatic tissue in the liver and led to accelerated HCC growth, which, in turn, resulted in a life span of only 58 days. These results highlight the importance of p53 in controlling genomic stability and carcinogenesis, likely via its powerful antioxidant mechanism.

Thorgeirsson and colleagues also showed [44] that activation of *c-MYC* is required to reprogram adult hepatocytes into hepatic cancer stem cells (CSCs), which promotes malignant progression. Moreover, c-MYC expression levels are determinant for the activation of CSCs in liver tumorigenesis. Thus, low c-MYC expression levels lead to increased proliferation and activation of CSCs, including the expression of markers associated with reprogramming (e.g., NANOG, OCT4 and EpCAM), expansion of side populations and acceleration of tumor growth [32,45]. However, if c-MYC exceeds a threshold, the potential for apoptosis and loss of CSCs occurs both in vitro and in vivo. Mechanistically, c-MYC induces the self-renewal capacity of liver CSCs in a p53-dependent manner. In fact, low c-MYC activation increases spheroid formation in p53-deficient tumor cells, while p53-dependent effects are blocked in the absence of c-MYC overexpression. Altogether, these results suggest a role for *c-MYC* in HCC development and establish a new gatekeeper role for p53 in repressing the c-MYC-induced CSC phenotype in liver cancer cells [44].

**Figure 1:**
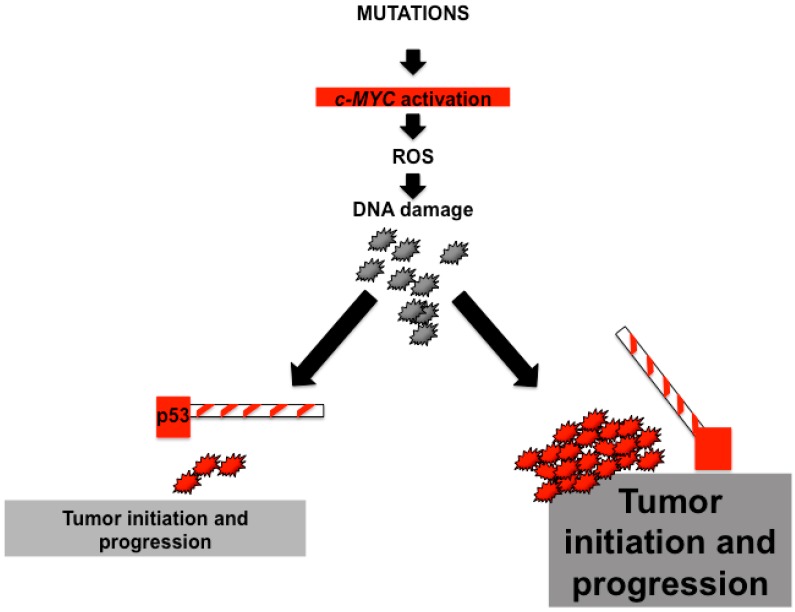
Cooperativity of c-MYC and p53 during tumor progression. Mutations that activate c-MYC, including amplification or translocation, cause reactive oxygen species (ROS) production and induce DNA damage. Absence of p53 (right panel) may override arrest responses, improve survival and drive cells with DNA damage into cycle. These redundant effects might trigger genome destabilization and acceleration of tumor progression.

Overall, the interactions between *c-MYC* and p53 are complex but extremely important for liver carcinogenesis. Mutations that activate c-MYC may generate sufficient ROS to induce DNA damage. In fact, most cells entering the cell cycle with DNA damage are expected to die mainly due to cellular responses orchestrated by p53. In contrast, an additional mutation, that completely disables the p53 pathway, improves survival and drives some cells with DNA damage into cell cycle. This situation creates a tolerant environment in which cells with *c-MYC* activation can both drive cell proliferation and induce chromosomal abnormalities [46]. These observations support the concept that p53 loss attenuates MYC-induced tumorigenesis accelerates tumor initiation and progression (Figure 1).

Several reports have shown the deleterious effect of several hepatotoxins on hepatic tumorigenesis. Non-genotoxic hepatotoxins induce cell proliferation and enhance tumor formation in the liver. Both 3,5-diethoxycarbonyl-1,4-dihydrocollidine (DDC) and carbon tetrachloride (CCl_4_) interact with c-MYC, and substantially accelerate the onset of HCC. Specifically, DDC- or CCl_4_-treated c-MYC mice display tumors between 31 and 40 days, respectively, compared to non-treated c-MYC mice in which the tumor burden occurs at 183 days. Altogether, these data indicate that the model of accelerated HCC occurs as a net result of changes in the liver very likely through transcriptional activation of Cyclin B1 [29].

A wealth of studies has investigated tumorigenesis using c-MYC^tg^. However, is this experimental murine model of HCC a faithful and reproducible model of human HCC? Thorgeirsson’s group analyzed and compared the gene expression pattern of HCC from different experimental mouse models of liver cancer and human HCC. They found that the gene expression pattern in HCC from c-MYC^tg^ and c-MYC/E2F1^tg^ transgenic mice were very similar to those with a better prognosis and survival rates in human HCC, while the expression pattern of HCC tissue from c-MYC/TGFα transgenic animals were closely associated with the poor survival group of human HCC patients [47]. Thus, the c-MYC^tg^ mice may represent, albeit with limitations, an interesting model to study human HCC.

## 4. Of Mice and Men—Is c-MYC Relevant for Liver Cancer?

*c-MYC* is associated with more than more than 70% of cancers [48,49]. In HCC, *c-MYC* was one of the first oncogenes identified for its high expression levels [50]. According to genetic analyses, overexpression of *c-MYC* is commonly caused by genomic amplification at 8q24.1 and present in up to 70% of viral and alcohol-related HCC [51]. Moreover, gains of 8q22-24 are among the earliest genomic events associated with liver cancer development [52].

Additionally, these genetic studies have revealed that *c-MYC* amplification is often observed in young patients, and in large and non-differentiated liver tumors. Indeed, c-MYC overexpression positively correlates with high proliferative activity and p53 levels. Besides, *c-MYC* is an indicator of poor prognosis in liver cancer, whereas disease-free survival in patients showing *c-MYC* overexpression is significantly shorter [50,53,54]. Furthermore, high c-MYC expression is also detected in metastatic and in recurrent hepatic tumors compared with primary HCC [55].

Moreover, HCC is a multi-stage process in which tumor development is caused by the accumulation of multiple genetic and epigenetic alterations. In fact, *c-MYC* signature has been linked with malignant conversion of pre-neoplastic hepatic lesions [56]. The amplification of the 8q22-24 region is found in 40–60% of early HCC, however, it is only observed in a small percentage of dysplastic nodules [57].

## 5. Treating Liver Cancer: From Theory to Practice

Taken into account the importance of *c-MYC* in HCC development, it represents an obvious target for novel therapeutic strategies for patients with HCC. The first evidence that c-MYC down-regulation might be used as a therapeutic approach to treat liver cancer was obtained from in vitro experiments. Simile and colleagues [58] evidenced that *c-MYC* down-regulation is capable of inhibiting cell cycle activity and growth of both human HepG2, and rodent Morris 5123 liver cancer cells. Several studies indicated that *c-MYC* inactivation can trigger senescence programs in primary HCC cell lines [59]. Interestingly, *c-MYC* blockade reverses hepatic tumorigenesis also in vivo. Qu and colleagues [60] recently evaluated temporal *c-MYC* disruption using the albumin promoter (SA)-expressing Cre-mutant estrogen receptor-2 (ERT2) system that yields hepatocyte-specific expression and activation of Cre recombinase induced by tamoxifen. The authors found that disruption of *c-MYC* in hepatocytes suppressed proliferation of liver parenchymal cells induced by a peroxisome proliferator-activated receptor-alpha (PPARα) agonist, Wy-14,643. Moreover, mice with hepatocyte-specific *c-MYC* disruption are also resistant to DEN-induced HCC, reinforcing the essential role of *c-MYC* in hepatocellular proliferation and tumor development [60].

Despite the complexity of genetic and epigenetic alterations in liver cancer, emerging evidence supports the interesting concept of “oncogene addiction”—first introduced to describe the dependency of some cancers on a single or a few genes, for the maintenance of their malignant phenotype and survival [61]. This concept is based on the fact that cancer might be “addicted” to *c-MYC*; whereby *c-MYC* inactivation restores the normal cellular checkpoint, resulting in: Proliferative arrest, apoptosis, cellular senescence, remodeling of the microenvironment and liver tissue architecture and suppression of vascular neoangiogenesis. However, the type of cancer strongly influences the specific effect of *c-MYC* inactivation [19]; while deregulated expression of *c-MYC* can contribute to genomic instability via the induction of reactive oxygen species (ROS) [62,63,64,65,66].

In the seminal work of Shachaf et al. [67], it became clear that c-MYC inactivation is sufficient to continuously stop invasive hepatic cancer, resulting in en masse differentiation of tumor cells into hepatocytes and biliary cells. Many of these tumor cells remained dormant as long as c-MYC remains inactivated; however, c-MYC reactivation immediately restored malignancy and invasiveness in these neoplastic cells. Next, the precise threshold level of c-MYC expression necessary for the maintenance of the tumor phenotype in T-cells was uncovered [68]. However, no analogous work was performed regarding HCC, something that will require intensive efforts in order to elucidate the role of *c-MYC* under these circumstances. Altogether, these findings support the hypothesis that continuous activation of signaling cascades associated with cell proliferation and survival is pivotal from the early stages of liver tumorigenesis. By targeting these pathways, the malignant phenotype of the cells could be reversed.

A druggable target—surprisingly, a tumor suppressor—that partners with c-MYC and enables its activity in p53-mutant liver cancer was found in a later study by Dauch and colleagues [69]. By using a direct in vivo small hairpin (shRNA) screening, they found that hepatic cancer cells with mutations in the gene encoding p53, driven by the oncoprotein RAS, became “addicted” to c-MYC stabilization through an aurora kinase A (AURKA)-dependent mechanism. c-MYC stabilization enabled cancer cells to overcome AURKA and p19^ARF^-mediated G2/M latency. The treatment of cells with allosteric inhibitors of AURKA kinase activity (MLN8237 and CD532) prevented the formation of new AURKA-phosphorylated c-MYC complexes, inducing c-MYC degradation and cell death.

Experiments using AURKA inhibitors in vivo and in vitro successfully suppressed RAS-driven c-MYC-expressing tumor growth and prolonged survival in p53-deficient mice. These findings suggest that interaction with AURKA might stabilize c-MYC, thus promoting entry into the cell cycle in the absence of functional p53. Therefore, conformational AURKA inhibitors that block this interaction may be a way to treat patients with c-MYC-positive, RAS-driven, p53-mutant liver cancer [69].

D’Cruz and colleagues [70], however showed that tumors lacking RAS mutations fully regressed following c-MYC de-induction, whereas tumors bearing RAS mutations did not, suggesting that secondary mutations in RAS contribute to tumor progression, thus pointing to the fact that c-MYC tumorigenesis proceeds through a RAS-dependent mechanism.

c-MYC interacts with Max—a common c-MYC partner protein—to form heterodimers that bind to DNA and induce transactivation. Disruption of the c-MYC-Max tetramer causes loss of function in all subsequent downstream target genes, suggesting that the c-MYC-Max interaction might be a very interesting molecular therapeutic target for cancer therapy [32,71,72,73,74,75,76]. We should note however that c-MYC might also regulate transcription, even in the absence of functional Max [77,78].

Additionally, thioxothiazolidinone, 10058-F4, is among the first small-molecule compounds which disrupt the interaction between c-MYC-Max and prevents c-MYC-target gene transactivation. In HepG2 cells, 10058-F4 arrested the cell cycle (at G_0_/G_1_ phase), induced cell death and significantly decreased alpha-fetoprotein (AFP) levels, an indicator of the extent of cellular differentiation. Moreover, 10058-F4 also downregulated human telomerase reverse transcriptase expression and abrogated telomerase activity [79]. Unfortunately, 10058-F4 IV administration in human prostate cancer-bearing mice has been disappointing, likely due to its rapid pharmacological properties, and to the fact that only low concentrations reach the tumors [80], rendering the drug ineffective. Hopefully, newer analogs will improve its pharmacokinetic features, including higher in vitro potency and lower metabolic lability [81].

Consequently, another small compound Quarfloxin (CX-3453), an inhibitor of c-MYC, is currently in phase II clinical trials (Cylene Pharmaceuticals) for the treatment of low to intermediate grade neuroendocrine carcinomas [82]. Although further investigations need to address the potential use of this compound in HCC; it remains a promising therapeutic option.

Even though c-MYC inhibitors are now being tested in the clinics, there are still possible limitations. First, c-MYC is a pleiotropic transcription factor, pivotal for normal cell proliferation and the maintenance of stemness. Nevertheless, the architecture of c-MYC-deficient hepatocytes is disorganized. Therefore, further investigations are crucial to determine whether c-MYC inhibitors may trigger side-effects. Second, c-MYC reactivation leads to tumor recurrence in transgenic mice, indicating that this experimental approach might target more mature cancer cells, rather than CSCs. Therefore, in order to enhance anti-cancer effects, combination with other therapeutic strategies such as chemotherapy might be essential [54,83].

Another critical model for MYC-mediated gene repression is through its ability to activate microRNAs (miRNAs) [84,85]. miRNAs are a diverse class of highly evolutionally conserved small RNAs that regulate the transcription and translation of genes [86]. Recently, a specific function of miRNAs in the pathogenesis of HCC has attracted considerable interest. Therapeutic strategies based on modulation of miRNA activity hold great promise due to the ability of miRNAs to influence cell behavior. Interestingly, Kota and colleagues [87] demonstrated the efficacy of miRNA-based therapy for HCC. A potential therapeutic miRNA should be expressed at low levels in tumor but highly expressed, and thus tolerated, in normal tissue. As an example, miR-26a fulfills these criteria. It is highly expressed in adult liver tissue but it is almost undetectable in human and murine liver cancer. Consistently, miR-26a induces cell cycle arrest at G1 in human liver cancer cells in vitro. Adenoviral administration of miR-26a in c-MYC-induced mice with HCC resulted in disruption of cancer cell proliferation, induction of tumor-specific apoptosis and protection from chronic liver disease. However, miR-26a does not directly target c-MYC in the described model. Still the notion that miRNAs may be useful as anti-cancer agents due to their ability to broadly modulate cancer cell proliferation and survival is highly attractive. Moreover, the treatment of tumors with c-MYC dysregulation is a paradigm closely related to clinical scenarios where such therapies could be employed [87].

Hence, there are distinct miRNA profiles characterized in liver tumors induced by distinct oncogenes including c-MYC. In a recent study by the group of Xin Chen [88], some miRNAs whose overexpression is capable of either delaying or abolishing tumor development in aggressive mouse models of liver cancer were identified. MiR-101 effectively prevented c-MYC and AKT/Ras-induced liver tumor development, providing strong evidence that miR-101 may be an ideal, innovative and effective therapeutic candidate against HCC.

Finally, c-MYC transcriptional function was also targeted by means of disrupting chromatin-dependent signal transduction [89]. Members of the bromodomain and extraterminal (BET) subfamily of bromodomain proteins associate with acetylated chromatin and facilitate transcriptional activation [90]. Filippakopoulus and colleagues first reported the development and biochemical characterization of a potent, selective small-mollecule inhibitor of BET bromodomains, JQ1. Later, the group of Bradner and Mitsiades reported the use of this drug for the modulation of c-MYC transcriptional activation [91]. These data indicate that these drugs can deplete c-MYC levels and thus down-regulate the c-MYC-derived transcriptional program, leading to cell cycle arrest and cellular senescence.

## 6. c-MYC: Causing Liver Stiffness

Liver fibrogenesis and end-stage cirrhosis are associated with the accumulation of extracellular matrix (ECM) proteins in response to acute or chronic liver injury from a wide array of etiologies including non-alcoholic steatohepatitis (NASH), non-alcoholic fatty liver disease (NAFLD), viral hepatitis and alcoholic liver disease (ALD). A highly-orchestrated interplay among parenchymal and non-parenchymal hepatic stellate cells takes place during fibrogenesis. Thus, hepatocyte death in response to exacerbated compensatory proliferation due to chronic liver insult is accompanied by the release of pro-fibrotic signals and the recruitment of infiltrating cells, which eventually lead to the activation of hepatic stellate cells (HSC). In response, HSC start to proliferate, produce alpha-smooth-muscle-actin (α-SMA) and deposition of ECM proteins such as collagen occurs [92,93].

Recently, we showed [94] that overexpression of c-MYC is frequently detected in patients with advanced stages of liver fibrosis and in experimental models of hepatic fibrosis. Furthermore, alb-Cre^+^/c-MYC^tg^ mice display spontaneous collagen deposition and early onset of hepatic fibrogenesis following chronic damage. Mechanistically, we observed that modulation of c-MYC in hepatocytes, either due to gene amplification or to the inflammatory response to liver injury triggered moderate hepatocyte apoptosis, increased hepatocyte proliferation and aberrant expression of platelet-derived growth factor subunit B (PDGF-B). This can be explained to the close physical vicinity of dying PDGF-expressing hepatocytes with resident quiescent HSC, which leads to HSC pre-activation and transdifferentiation into myofibroblasts, resulting in ECM accumulation. The “priming” effect on HSCs triggers high ECM production/deposition, especially, after a second pro-fibrotic hit. Therefore, c-MYC overexpression may be used not only as a novel marker for liver fibrogenesis in mice and men, but also as a co-adjuvant tool for the improvement of diagnosis in patients.

Cirrhosis often leads to liver cancer [95]. Indeed, the mechanism of the progression from cirrhosis to HCC remains poorly investigated. A previous report indicated the strong link between cirrhosis, HCC and the gene modification and methylation state of *c-MYC* in human liver tissue. In fact, hypomethylation of the c-MYC gene has been linked with liver cancer development [96], indicating that the state of DNA methylation can be used as a diagnostic tool for chronic liver damage.

Given the strong link between chronic liver injury, hepatic fibrogenesis and HCC development in humans, novel experimental models are needed in which HCC can be induced within the microenvironment of liver fibrosis. A very interesting method was reported in a recently, where the authors used hydrodynamic transfection coupled with the Sleeping Beauty transposase system [97]. In order to induce HCC within the context of hepatic fibrosis, mice were hydrodynamically transfected with c-MYC-expressing transposomes and short hairpin RNA (shRNA) down-regulating p53 (shp53). Treatment with CCl_4_ alone did not induce tumorigenesis, but promoted c-MYC and shp53-induced HCC, leading to tumor development within three months of treatment. Altogether, these data draw a very interesting conclusion: liver fibrogenesis significantly accelerates HCC induced by c-MYC overexpression and p53 repression.

To sum up, persistent elevation of c-MYC expression in cirrhosis contributes to HCC development [98]. In the need for redefining the function of c-MYC in liver fibrogenesis, cirrhosis and end-stage HCC, it is necessary to explore pathways regulated by c-MYC and, ultimately, link these data with clinical outcomes and therapeutic strategies.

## 7. c-MYC and Viral Hepatitis: Playing the Same Tune

**Figure 2:**
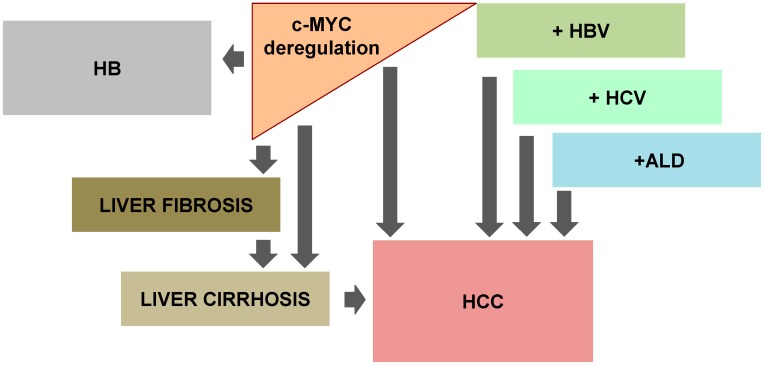
Deregulation of c-MYC and liver disease. Deregulation of c-MYC expression has been observed in hepatoblastoma (HB), liver fibrosis and cirrhosis, and hepatocellular carcinoma (HCC). In addition, c-MYC function plays an essential in HCC development due to alcoholic liver disease (ALD) and viral hepatitis (HBV and HCV).

Approximately 80% of the cases of HCC are associated with cirrhosis related to chronic hepatitis B virus (HBV) or hepatitis C virus (HCV) infections [95]. Several sub-genomic protein fragments of HBV, namely X, S and pre-S, have transactivation properties, but efforts have concentrated on the X-protein (Figure 2).

The hepatitis virus B X protein (HBx) transforms hepatocytes through multiple mechanisms. *c-MYC* is a critical target gene activated by HBx. In turn, activation of c-MYC accelerates HBx-mediated oncogenic properties [54,99]. Several experimental models support this notion. An in vivo study by Teradillos and collaborators evidenced that co-expression of the HBx and *c-MYC* transgenes accelerated HCC development in transgenic mice. Thus, in this experimental model, HBx alone has no direct pathological effects; however, it accelerates c-MYC-induced tumor development. Average tumor latency was reduced by two to three months in c-MYC/HBx mice compared to single c-MYC transgenic animals. Altogether, the evidence that co-expression of the HBx and *c-MYC* transgenes accelerated HCC development in transgenic mice clearly establishes this viral transactivator as a tumor promoter, and as a cooperating partner of the *c-MYC* oncogene in liver cell transformation [30].

In another study, a model of transgenic mice incorporating the region encoding amino acids 58–154 of the HBx protein and the murine *c-MYC* gene was used. This model also showed changes in the liver after birth with dysplastic foci evolving into nodules and overt HCC between weeks 20–28 of age. The entire process illustrates the impairment in cell growth and death caused by the synergistic effect of HBx and *c-MYC* that trigger the development of liver cancer after a prolonged period of latency. Further support of this two-hit theory was obtained with the HBx gene, which provides the triggering stimulus, and factors like the *c-MYC* protoncogene complete the malignant transformation [100]. Moreover, HBx increases c-MYC stability by inhibiting the SCFSkp2 ubiquitin E3 ligase-mediated c-MYC ubiquitination. This stabilization greatly contributes to viral oncogenesis [101].

The analogous situation is associated with HCC development in humans. The analyses of human liver revealed that *c-MYC* amplification occurs more frequently in young patients who have HBV infection [50]: Comparing molecular profiles showed that many of the genes regulated by *c-MYC* are involved in HBV-related HCC, thus supporting the notion that *c-MYC* is related to the oncogenic activity of HBV [102].

Moreover, HBV infection is the most epidemiologically associated risk factor for the early-onset HCC (patients younger than 40), which accounts for 15–20% of total liver cancer (only in Asia) with further increasing incidence. Given that the HCC development involves the interplay between HBV and host hepatocytes, both virus and human genomes may contribute to the pathogenesis, either individually or synergistically. Regarding the HBV factors, the HBV B2 genotype has been shown to strongly influence the clinical outcome, including cirrhosis and early-onset HCC. HBV integration is a common phenomenon, even in early-onset HCC, but the conditions between early- and late-onset diseases are rather different. A breakpoint between *c-MYC* and plasmacytoma variant translocation 1 (*PVT1)*, located in the 8q24 gene desert, is frequently detected in early-onset HCC, resulting in overexpression of *c-MYC* in tumors [103,104]. However, the stage in tumor development when this integration occurs, and how important it may be for HCC, is currently unknown. Thus, further studies are needed to shed light on the role of this integration site in HCC development. In order to develop a therapeutic strategy for HCC based on gene silencing, the effect of siRNAs on HBx and *c-MYC* gene expression and their transactivation functions has been thoroughly analyzed in vitro. Even though each siRNA showed different efficiency, the inhibitory effects of using two different siRNAs were cumulative. These results hold promise for the future development of siRNA-based therapy against HBV-induced HCC [105,106].

Chronic infection with HCV is another major risk factor for HCC development. In general, HCC develops only after two or more decades after HCV infection. However, HCV patients with cirrhosis or advanced fibrosis exhibit an enormous risk of developing HCC [107]. Oxidative stress and elevated ROS production are common mechanisms of HCV infection and play a pivotal role in HCV-associated HCC. Importantly, among other factors, the oncogene *c-MYC* can induce ROS production. HCV in hepatocytes increases c-MYC expression in in different scenarios: (i) Non-tumoral liver tissue of HCV-infected patients with or without HCC; (ii) In hepatocyte cell lines harboring an HCV replicon and the infectious HCV strain JFH1; and (iii) In an in vivo transgenic murine model expressing the complete HCV open reading frame (ORF) in a liver-specific manner [108].

Mechanistically, the activation of Akt by the HCV non-structural protein NS5A, and subsequent stabilization of β-catenin, have been suggested to be responsible for *c-MYC* activation and promoter transcription. Therefore, β-catenin-dependent c-MYC expression in this context leads to increased ROS production, mitochondrial disturbance, increased DNA damage and aberrant cell-cycle arrest. All these changes play a crucial role in HCV-associated oxidative stress and genetic damage, very likely contributing to HCV-related HCC [108].

## 8. Drinking and Thinking of c-MYC

Excessive alcohol consumption is the oldest form of liver injury known to civilization and currently remains a major cause of chronic liver disease throughout the world. ALD includes a spectrum of injury ranging from simple steatosis to liver cirrhosis, which eventually leads to end-stage HCC [109,110]. Case-control studies in different countries indicate that chronic ethanol consumption is associated with approximately twofold increased chance of developing HCC. Despite the fact that pathways causing alcohol-induced liver cancer are still poorly understood, many other factors other than the cumulative amount of alcohol ingested often determine the progression of alcohol-induced carcinogenesis [111,112]. For instance, only 35% of heavy drinkers develop advanced stages of ALD and 1–1.5% of patients with decompensated alcohol-induced cirrhosis have HCC [113,114]. Among these risk factors, endogenou*s* (i.e., genetic) factors are of major relevance [115,116,117]. The identification of such risk factors, which, synergistically with ethanol intake, drive alcoholic injury in the direction of cancer development, would have great benefits for the optimization of therapeutic strategies [117]. The detection of such pre-existing risk factors could be used to identify “high risk” individuals in whom preventive measures, such as counselling or physical examinations, should be undertaken. In this context, our recent work [118] clearly showed that liver overexpression of the oncogene *c-MYC* correlates with earlier induction and more dramatic progression of ALD and thus represents an endogenous risk factor.

Feeding hepatocyte-specific alb-Cre^+^/c-MYC^tg^ mice with the EtOH Lieber-DeCarli diet for four weeks results in impaired cell proliferation and early ballooning degeneration, increased pro-fibrotic signaling with hepatic collagen deposition, altered fat metabolism, generation of ROS and changes in mitochondrial morphology associated with energy dysfunction. Hence, long-term Lieber-DeCarli diet feeding of alb-Cre^+^/c-MYC^tg^ mice results in substantially elevated deposition of hepatic collagen and expression of pre-neoplastic markers. Moreover, we found that liver *c-MYC* is strongly up-regulated in patients with advanced ALD.

Taken together, our study showed for the first time that the degree of *c-MYC* expression is an essential mechanism in the pathophysiology of EtOH-derived chronic liver injury and highlighted c-MYC as a novel marker in patients to predict the risk of developing advanced ALD [118].

## 9. Notorious c-MYC: Involvement in Pediatric Liver Cancers

In adults, HCC is by far the most frequent form of liver malignancy. Indeed, in infants, the most common form of liver tumor is hepatoblastoma (HB), a tumor that mostly affects children less than three years of age, which comprises approximately 1% of all pediatric cancers (Figure 2).

HB is an embryonal tumor characterized by proliferation of immature hepatoblasts, frequently associated with malignant mesenchymal tissue; which suggests that it derives from undifferentiated progenitor cells [119]. Because HB develops in the absence of liver disease or viral infection, this type of tumor has a strong genetic footprint. Such as aberrant activation of the Wingless integrated (Wnt) signaling pathway is virtually universal, although generally not sufficient for tumorigenesis. Hence, few studies have implicated c-MYC and showed its cooperation with mutant β-catenin to drive HB [120,121]. To address the precise role of both oncogenes in the pathogenesis of HB, Comerford et al. [120] recently generated transgenic mice in which c-MYC and mutant β-catenin were targeted in immature cells of the developing mouse liver. Perinatal co-expression of both genes promoted the preferential development of HB over other tumor types in neonatal mice, mimicking the human disease. The authors elegantly proved that Wnt/β-catenin and c-MYC are potent, perhaps even “preferred” collaborators for HB development. They delineated the contributions of both oncogenes to HB development and identified *c-MYC* as the dominant determinant of the HB transcriptome [120].

In another study, the analysis of the excessive Wnt/β-catenin and c-MYC signaling pathways was performed in a large number of human tumor specimens and biopsies from 85 patients. Microarray analysis was performed and two tumor subclasses that evoke early and late phases of prenatal liver development were identified. The highly proliferating subclass was classified by gains of chromosomes 8q and 2p and upregulated c-MYC signaling. Major differences in expression profiles of these two HB subtypes elucidated a 16-gene signature that discriminated both invasive and metastatic HB, and patient prognosis, with high accuracy [121].

High-grade undifferentiated tumors such as HB are enriched in CSCs. miRNAs have been integrated recently into regulatory networks that control stem cell identity as well as tumor pathogenesis. The miRNA expression profiling of HB confirmed differential patterns associated with the developmental stage and the activity of c-MYC. Undifferentiated and aggressive HB overexpress the miR-371-3 cluster and display down-regulation of the miR-100/let-7a-2/miR-125b-1 cluster, eliciting the expression profile of a typical embryonic cell. Importantly, both miRNA clusters exert antagonistic effects on cell proliferation and tumorigenesis, and are regulated by c-MYC in different ways. Thus, the interplay between the two miRNA clusters strongly affects oncogenic processes, implicating stem cell-like regulation of c-MYC-dependent miRNAs in poorly differentiated HB. Consistently, targeting this regulatory circuit might be beneficial for the treatment of c-MYC-related HB [122].

## 10. Study in Yellow: c-MYC in Hepatic Cholestasis

Cholestatic liver injury remains a major cause of chronic liver disease with limited treatment options. In a very interesting series of publications, Yang and collaborators reported the possible roles of c-MYC and its antagonist MAX´s next tango (MNT) in chronic cholestasis. First, the researches unveiled a novel switch from MNT to c-MYC expression during cholestasis in vivo after bile duct ligation (BDL)—an experimental model of obstructive cholestasis—as well as after treatment of hepatocytes with a toxic bile acid [123]. This finding has important pathological implications as it leads to the induction of p53 and cyclin D1, which are actively involved in the pro-apoptotic effect of toxic bile acids [124]. Interestingly, c-MYC expression was induced early and persisted until the end-point in a combined model of cholestasis and diethylnitrosamine (DEN)-induced carcinogenesis. In functional studies, several mechanistic events, which accompany the induction of cholestasis that contribute to carcinogenesis, have been elegantly elucidated. This includes downregulation of miR-34a, upregulation miR-210, and replacement of MNT by c-MYC in the binding to cyclin D1. As a proof of principle, knockdown of *c-MYC* reduced carcinogenesis, while knockdown of MNT accelerated its progression [123,125].

In a very recent paper, it was shown that the interplay between c-MYC, MATα1 and MAF proteins, and their deregulation during chronic cholestasis may facilitate oncogenesis. This work provides strong evidence that c-MYC, MATα1, MafG, and c-Maf interact with each other directly. Remarkably, MAT1A overexpression, or c-MYC, MAFG, or c-MAF inhibition in tumor cells dramatically inhibited their in vitro and in vivo growth. By contrast, up-regulation of c-MYC, MAFG, and c-MAF or attenuation of MATa1 expression by knock-down triggered increased tumor cell growth and invasion in vivo [126,127].

Altogether, these studies enhance our understanding of the process by which cholestasis contributes to carcinogenesis, and identify potential targets for prevention of cancer in patients with cholestatic liver disease. It will be exciting to see whether future developments based on rational targeting of the molecules and pathways elucidated in this study will lead to improvement in the treatment [125].

## 11. Good News at the End

The liver has a pivotal role in glucose metabolism. If plasma glucose is high, the liver processes this excess and replenishes glycogen stores. During starvation, the liver releases glucose into the blood via glycogenolysis and gluconeogenesis [128]. Glucose transport and phosphorylation are the first stages of glucose utilization in the liver. Furthermore, glucose levels regulate gene transcription in the liver [129]. Several transcription factors, such as c-MYC, control glucose expression. The c-MYC transcription factor plays an important role in hepatic carbohydrate metabolism. Thus, liver glucose metabolism could, in fact, determine the blood glucose and insulin set points in c-MYC transgenic mice. Moreover, an elevation in c-MYC levels induces hepatic glucose utilization and accumulation [130]. After the glucose tolerance test, c-MYC transgenic mice exhibit lower levels of blood glucose than their control littermates, indicating that c-MYC overexpression results in an increase in blood glucose disposal by the liver. These data led to the hypothesis that the increase in c-MYC can prevent diabetic hyperglycemia. In order to support this notion, the group of Valera induced diabetes in c-MYC transgenic and control mice by short-term (seven days) streptozotocin (STZ) treatment, a compound that has a preferential toxicity towards pancreatic β-cells, leading to their destruction and absence of insulin. They clearly show that c-MYC overexpression in livers of transgenic mice prevented the development of diabetes following streptozotocin (STZ) treatment, not only by inducing hepatic glucose uptake and utilization, but also by blocking gluconeogenesis and ketogenesis [131].

Subsequently, these findings were fully proven in the long-term STZ model. After four months of STZ treatment, c-MYC overexpression in livers of transgenic mice partially prevented diabetic hyperglycemia in fed animals, as well as leading to normoglycemia during starvation. This was likely achieved through c-MYC’s function both to induce hepatic glucose uptake and utilization, and to block gluconeogenesis. Improvement in liver metabolism of STZ-treated transgenic mice also resulted in prolonged survival and maintenance of body weight [132].

Moreover, final validation came from a high-fat diet (HFD) model [133]. After three months on HFD, control mice developed insulin resistance, obesity, hyperglycemia and hyperinsulinemia. In contrast, c-MYC transgenic mice remained lean and showed improved glucose disposal as well as normal levels of blood glucose and insulin, indicating that they are protected against obesity and insulin resistance. These findings were concomitant with normalization of gene expression of hepatic glucokinase (HG), L-pyruvate kinase (LPK), sterol receptor element binding protein 1-c (SERBP1-c), peroxisome proliferator activated receptor alpha (PPAR-α), and uncoupling protein-2 (UCP-2) in the liver of transgenic mice fed with the HFD.

It is worth noting that Type I diabetes is the result of autoimmune destruction of the insulin-producing β-cells of the pancreas and is mainly characterized by lack of insulin, triggering the development of severe hyperglycemia. Therefore, increasing glucose uptake and utilization by liver can reduce diabetic hyperglycemia.

Altogether, these results using c-MYC overexpression reinforce the role of hepatic c-MYC in maintaining glucose homeostasis, and suggest that the increase of glucose uptake and utilization could be a useful therapeutic approach for the treatment of diabetes mellitus [133].

## 12. Final Considerations

*c-MYC* acts as a master regulator of cell growth and cell cycle arrest. In fact, *c-MYC* directly regulates genes involved in cell cycle regulation such as cyclin-dependent kinase-4 (CDK4), a well-documented c-MYC target [32,134,135]. Moreover, *c-MYC* plays an essential role during normal development—driving the expansion of transit amplifying cells. Emerging data suggests that deregulation of *c-MYC* function might be associated not only with HCC development, but also with chronic liver disease, such as ALD, viral hepatitis, liver fibrosis/cirrhosis and HB). Since deregulation of c-MYC expression is a very early event in liver carcinogenesis, c-MYC overexpression in hepatocytes has been extensively studied using transgenic mice. However, do these experimental models mimic human HCC development? The prognosis and survival rates of these mice are very close to the human situation, suggesting that they might be a suitable experimental approach. In fact, c-MYC overexpression, frequently caused by genomic amplification at 8q24.1, is associated with human HCC, validating the importance of transgenic mice models. Thus, c-MYC has become an attractive and plausible therapeutic target for chronic liver disease. Therefore, cancer might become “addicted to c-MYC”, which may warrant the use of c-MYC modulators in the future that can minimize or prevent undesirable side-effects, perhaps in combination with other therapeutic strategies.

In summary, the overwhelming evidence suggesting a crucial role for *c-MYC* in liver disease warrants further investigation utilizing transgenic mice models in combination with hepatotoxins, may lead to the discovery of novel functions of this ubiquitous transcription factor, and to novel therapeutic strategies for a broad range of hepatic injuries.

## Figures and Tables

**Table 1:** c-MYC induced HCC: lessons from transgenic mice.

Transgenic Mice	Phenotype	Time to Tumor Development	References
alb-Cre^+^/c-MYC^tg^ (hepatocyte-specific)	HCC development albeit with a long latency period	45 weeks of age—less than 40%; 65 weeks—80% of mice have a minimum of one liver tumor	[22,23,24]
alb-Cre^+^/c-MYC^tg^/TGFα^tg^ (hepatocyte-specific)	Dramatic acceleration of HCC neoplasia	17 weeks—20% with tumor 40 weeks—100% with tumor	[23,25,26]
alb-Cre^+^/c-MYC^tg^/HGFα^tg^ (hepatocyte-specific)	Delayed appearance of preneoplastic lesions and prevention of malignancy	12 months of age—15% of mice display dysplastic cells; 16 months—67% affected by mild hepatic dysplasia	[25]
alb-Cre^+^/c-MYC/^tg^E2F1^tg^ (hepatocyte-specific)	Aggressive tumor phenotype	26–35 weeks—100% of mice have tumors	[27]
alb-Cre^+^/c-MYC^tg^/p53^−/−^	Accelerated increase in size and malignancy of HCCs	250 days—100% moribund with tumor burden	[28]
alb-Cre^+^/c-MYC^tg^/IgEGF^tg^/p53^−/−^	Accelerated HCC growth	58 days—100% moribund with tumor burden	[28]
c-MYC^tg^ tetracycline-regulated, liver specific) + DDC	Accelerated HCC	31 days—100% moribund with tumor burden	[29]
c-MYC^tg^ (tetracycline-regulated, liver-specific) + CCl_4_	Accelerated HCC	40 days—100% moribund with tumor burden	[29]
WHV/c-MYC/HBx	Accelerate tumor development	60 weeks—100% mice developed large tumors	[30]

Hepatocellular carcinoma (HCC); Transforming growth factor-alpha transgenic mice (TGFα^tg^); Hepatocyte growth factor-alpha transgenic mice (HGFα^tg^); Hepatitis virus X protein (HBx); Carbon tetrachloride (CCl_4_); Wood-chuck hepatitis virus (WHV); Diethyl 1,4-dihydro-2,4,6-trimethyl-3,5-pyridinedicarboxylate (DDC); Epidermal growth factor (EGF).
